# Gastrointestinal Tolerance of Short-Chain Fructo-Oligosaccharides from Sugar Beet: An Observational, Connected, Dose-Ranging Study in Healthy Volunteers

**DOI:** 10.3390/nu14071461

**Published:** 2022-03-31

**Authors:** Cindy Le Bourgot, Florian Rigaudier, Christine Juhel, Florent Herpin, Claire Meunier

**Affiliations:** 1 Tereos, R&D Department, 77230 Moussy-le-Vieux, France; meunierclaire@yahoo.fr; 2 CEN (CEN Nutriment Unit), 21000 Dijon, France; florian.rigaudier@groupecen.com (F.R.); christine.juhel@groupecen.com (C.J.); florent.herpin@groupecen.com (F.H.)

**Keywords:** prebiotics, dietary fibres, nutrition, gut health, healthy humans, digestive tolerance, sugar beet fibre

## Abstract

Dietary fibres are important in the human diet with multiple health benefits. This study aimed to determine the gastrointestinal tolerance of short-chain fructo-oligosaccharides (scFOS), well-known prebiotic fibres, at doses up to 40 g/d. An observational, connected, dose-ranging trial was conducted in 116 healthy volunteers. During the first week, the participants were instructed to consume their usual diet. During the second week, half of the subjects consumed 15 g scFOS per day, and the other half consumed 20 g scFOS per day. For the third week, the scFOS dose was doubled for all subjects. Gastrointestinal symptom severity was reported daily, as well as stool consistency and frequency. The results show that scFOS are well tolerated up to 40 g/d; all reported symptoms remained very mild from a clinical perspective. Stool consistency stayed normal, between 3 and 5 on the Bristol stool scale, confirming that no diarrhoea appeared after scFOS intake. Stool frequency also remained within the normal range. In conclusion, scFOS intake is well tolerated up to 40 g/d in healthy subjects. Thanks to their short chains and unique composition, scFOS prebiotic fibres are much better tolerated than other types of inulin-type fructans with longer chains. The digestive tolerance of fibres should be considered when added to foods and beverages.

## 1. Introduction

Dietary fibres are important parts of the human diet and have multiple physiological effects that are beneficial to health. They are well known for their beneficial effects on bowel function, such as improving regularity, but also beyond gut health [1]. Indeed, epidemiological and clinical studies have demonstrated that the intake of dietary fibres, known for their promotion of more diverse and balanced intestinal microbiota, is inversely associated with metabolic disorders such as obesity and type 2 diabetes [2,3]. Dietary fibre intakes in adults across Europe are estimated to be 16 to 24 g per day, with little variation from one European country to another [4]. Although consumers are often aware of the beneficial health effects of dietary fibre, many do not consume enough. Indeed, the current average fibre intake is insufficient to meet the needs of the body and is below the recommendations. For the general adult population, the European recommendations for fibre intake are at least 25 g per day, with a satisfactory intake at 30 g per day [5].

Dietary fibres are defined in the EU regulation 1169/2011 on the provision of food information to consumers as ‘carbohydrate polymers with three or more monomeric units, which are neither digested nor absorbed in the human small intestine and which have a beneficial physiological effect demonstrated by generally accepted scientific evidence’. There are different types of fibres, and according to their nature (naturally occurring in food, obtained from food raw material, or synthesized), physical properties, and fermentability in the gut, they do not have the same benefits for the consumer [6]. The beneficial effects associated with high-fibre diets are linked to their lower caloric value (2 kcal/g vs. 4 kcal/g for digestible carbohydrates), and their lower postprandial glucose excursion [7]. In addition, some dietary fibre exerts fermentative activity in the gastrointestinal microbiome. Prebiotics are defined as nondigestible food ingredients that beneficially affect the host by selectively stimulating the growth and/or activity of one or a limited number of bacterial species already resident in the colon, and thus attempt to improve host health [8]. 

Short-chain fructo-oligosaccharides (scFOS), composed of a terminal glucose molecule linked to fructose molecules by a β1–2 bound with a low degree of polymerisation (DP 3–5), are one of the most studied fermentable dietary fibres. Other well-known fermentable dietary fibres are inulin, extracted mainly from chicory roots, with a DP varying from 2 to 60, and oligofructose produced from inulin by partial enzymatic hydrolysis with a DP 2 to 9. Short-chain FOS are obtained from sucrose (beet or cane) after enzymatic reaction. They are frequently used in a series of food and beverages, for fibre enrichment, which is nutritionally relevant given the low intake of fibre in some countries. They can also contribute to reducing food sugar content and energy [9]. The effects of scFOS consumption have been extensively studied in healthy adults, showing they are selectively fermented by some bacteria, especially Bifidobacteria, in a dose–response manner for doses ranging from 2.5 to 20 g/day. Positive effects have been demonstrated on the colonic environment and digestive comfort [10,11]. A regular consumption of a low dose of scFOS at 5 g per day, diluted in a drink or mixed with solid food for 6 weeks, significantly increased the number of bowel movements, helping to fight constipation issues [12]. In addition, a recent meta-analysis demonstrated that short-chain β-fructans with a degree of polymerisation lower than 10, therefore including scFOS, improve stool frequency and consistency [13]. Such an effect was not true for longer-chain fructo-oligosaccharides such as inulin.

Dietary fibres are usually well tolerated but may also have some dose-related undesirable effects due to their natural osmotic potential and excessive fermentation. There is significant intrasubject variability depending on the characteristics of dietary fibres, notably their degree of polymerisation. Digestive tolerance is currently assessed by recording the gastrointestinal symptoms commonly observed after fibre consumption, like flatulence, borborygmi, bloating, and abdominal cramps [14]. It is difficult to distinguish between an acceptable and a non-acceptable side effect of fermentation. Flatulence, for instance, is a well-known and often accepted side effect of fibre consumption. Stool frequency and stool consistency records provide additional data on laxation, with particular attention on diarrhoea. Stool softening may be a desired effect; however, diarrhoea is clearly a non-acceptable symptom.

The availability of scFOS as an additional source of dietary fibres can increase the fibre intake to a level that is closer to the recommended daily value. There is no doubt that scFOS are safe, because they are consumed regularly as part of the normal diet up to 20 g/d without any deleterious effects [10,15] and numerous clinical and pre-clinical studies have already demonstrated the multiple beneficial effects of dietary fibres on health [13,16,17]. However, it is crucial to determine the gastrointestinal tolerance and the global acceptance of this product at higher dosages to be able to quantify the maximum level of possible fibre enrichment of diverse products offered to consumers to help them to reach the European nutritional recommendations. Only one study evaluated the digestive tolerance of a high consumption of scFOS in healthy adults [18], but this study is quite old and was conducted with a small population, with a gradual daily increase of the dose of scFOS. The dietary and lifestyle habits have significantly evolved over the past decades, which could impact the digestive tolerance of this kind of product. Studies conducted in other types of prebiotic fibres tested the digestive tolerance up to 20 g/d, sometimes with some deleterious gastrointestinal effects [19,20,21]. In addition, the digestive tolerance seemed different depending on the type of oligosaccharides and their chain length or DP, with shorter-chain oligosaccharides being better tolerated than longer ones [22], leading to questioning of the digestive tolerance of each prebiotic fibre.

This study aims to evaluate the gastrointestinal tolerance of scFOS consumed on a daily basis by healthy volunteers, during 2 weeks at different doses, from 15 to 40 g.

## 2. Materials and Methods

### 2.1. Subjects

A total of 116 healthy French volunteers were recruited in this study after solicitation via electronic form by CEN Nutriment. The subjects were males and females, aged from 18 to 50 years, with a regular stool frequency (≤3 stools per day or ≥3 stools per week, according to Rome IV criteria) during the 2 weeks preceding inclusion, wishing to maintain their hygienic and dietary habits during the study (food and physical activity), and having a smartphone compatible with the Nurstrial^®^ data collection application dedicated to subject-reported outcomes. The main exclusion criteria were: regular gastrointestinal symptoms or ongoing or chronic gastrointestinal pathology, antibiotic treatment received in the past two months, woman with dysmenorrhea (abdominal pain), use of a food supplement or drugs having an action on intestinal transit (based on fibre, prebiotics or probiotics, osmotic laxatives, etc.), diet with a high- or low-fibre intake according to the Programme National Nutrition Santé (PNNS) score, pregnant or breastfeeding woman, food allergies or intolerances, gallstones presence, and being involved in another study or have been in the last two months. All subjects gave their informed consent before participating in the trial.

### 2.2. Study Design and Settings

The study was an observational study using a connected model, and each subject was required to complete the survey on the Nurstrial^®^ secured application via their smartphone. A favourable ethics opinion from an Independent Ethics Committee (IRB) was issued dated 27 August 2020 (opinion 2020/CE 64; Clermont-Ferrand). The study took place from November 2020 to March 2021.

The gastrointestinal tolerance of scFOS from sugar beet (Actilight^®^ P95; degree of polymerisation (DP) 3-5; Beghin-Meiji) was compared to a baseline period without supplementation. Actilight^®^ scFOS is a unique prebiotic fibre, obtained from sugar beet sucrose through an enzymatic reaction, with a proprietary fructofuranosidase enzyme, leading to very short-chain structure (degree of polymerisation between 3 and 5). It is composed of a terminal glucose molecule (G) linked to fructose molecules (F) by a β1–2 bound with a consistent and guaranteed composition, with 37 ± 6% 1-kestose (GF2), 47 ± 6% nystose (GF3), and 16 ± 6% 1F-β-fructofuranosyl nystose (GF4), thanks to high-purity raw materials and a strictly monitored process.

**Figure 1:**
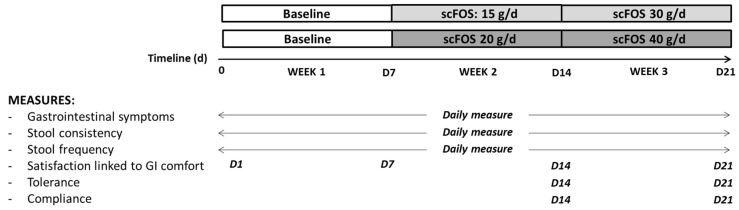
Study design.

The subjects were divided into two groups in a non-random order with a recruitment in favour of a 50/50 male–female distribution in each group. In the two groups, the subjects had to follow their usual diet for the first week of the study (week 1). In the first group, the subjects consumed 1 sachet of 15 g of scFOS for 7 days (week 2) and 2 sachets of 15 g of scFOS during the last 7 days of the trial (week 3). In the second group, the subjects had to take one sachet of 20 g of scFOS during week 2 and 2 sachets of 20 g during week 3. The contents of each sachet were dissolved into water or added to a yogurt and consumed preferably in the middle or at the end of the meal. During the third week of the trial, scFOS was consumed daily in two separate doses (one sachet at lunch and another one at dinner). For both groups, the total study period for each subject was three weeks (Figure 1).

At the first connection to the Nurstrial^®^ application and before consumption of the tested product, the subjects followed the instructions and filled in their sociodemographic characteristics (sex, age, weight, height), number of stools per week during the last two weeks, and satisfaction with gastrointestinal comfort over the past two weeks. 

During the whole study, the subjects were asked to maintain their standard diet and to declare any significant modifications to their daily diet. Any significant modifications to the food regimen, concomitant medications, or physical activity habits during the study were recorded and invoked to eliminate the subject on a case-by-case basis during the data review.

### 2.3. Course of Study and Gastrointestinal Tolerance Assessment

On the first day of inclusion, the subjects completed data about any gastrointestinal symptoms experienced during the day as well as the stool frequency and consistency of the day. The subjects also completed PNNS food frequency and GPAQ physical activity questionnaires [23].

During each of the 21 days of the study, the subjects had to complete daily, at the end of the day, data concerning gastrointestinal symptoms, stool frequency, and stool consistency. The primary outcome of the study was the change in gastrointestinal comfort experienced between the 7 days of baseline without consumption of scFOS and the 14 days of consumption of scFOS. The severity of eight solicited symptoms was recorded daily on the Nurstrial^®^ application: bloating, borborygmi, flatulence, abdominal pain, nausea, vomiting, heartburn, and acid reflux. The severity was assessed using a Likert scale with 4 items: 0 corresponding to ‘no symptom’ and 3 corresponding to ‘severe symptom’.

In addition, throughout the study, the stool consistency was collected daily on the Bristol stool scale (BSS), a diagnostic tool to evaluate samples of human faeces based on the shape and consistency of the stool [24]. The stools were assigned a number from 1 to 7 that corresponds to descriptions on the scale: type 1 for ‘separate hard lumps’, and type 7 for ‘entirely liquid, watery, no solid pieces’. Stool frequency was recorded daily throughout the study.

Finally, at days 7, 14, and 21, the subjects had to indicate their satisfaction linked to gastrointestinal comfort by using a Likert scale with 5 items: 1 for ‘not at all satisfied’ to 5 for ‘very satisfied’. At days 14 and 21, the general tolerance of the product through the declaration of non-GI inconvenience was also reported, as well as the number of remaining sachets, respectively, from week 2 and week 3. Finally, at day 21, the subject was required to complete the PNNS and GPAQ questionnaires again, considering the potentially confounding factors of intestinal transit and gastrointestinal symptoms. The PNNS food frequency questionnaire is based on 12 questions related to food habits, as described by Estaquio et al. (2009) [25]. The first 8 questions correspond to a category of food consumed, ranging from the consumption of starches to that of fish, and the subject has the choice of answering on a scale of 1 to 7 or 8: 1 corresponding to a consumption of 4 times or more per day, and 7 or 8 (depending on the question) corresponding to never consumed. Questions 9, 10, and 12 correspond to their consumption of fats, sugary products, and salt, with a choice of answers between 1 and 4: 1 for very high and 4 for insufficient consumption. Question 11 corresponds to fluid consumption, with 1 for only water and 4 for only other drinks. The GPAQ physical activity questionnaire is based on activities at work, movement from one place to another, and hobbies, as described by Widad (2016) [23]. This questionnaire considers the total time spent exercising in a typical week, the number of days of physical activity, and the intensity of physical activity. Then, three levels of physical activity are proposed to classify the populations: low, moderate, and intense physical activity.

### 2.4. Statistical Analysis

The analysed population was composed of subjects respecting the inclusion and exclusion criteria, having taken a sufficient and assessable amount of the product under study, and whose main criteria were evaluable, meaning sufficient data for each phase of the study was obtained. This was assessed on a case-by-case basis during the data review. The product tolerance was evaluated on subjects who consumed the product at least once.

Comparisons of the means were made using T-tests and analysis of variance (ANOVA) with repeated measures. Percentage comparisons were made using the Chi-squared test (or Fisher’s exact test if the conditions for applying the Chi-squared test were not met). Statistical analysis was realised on SAS software (version 9.4; SAS Institute Inc., SAS Campus Drive, Cary, NC, USA) and a difference was defined as significant with a *p*-value ≤ 0.05.

## 3. Results

**Figure 2:**
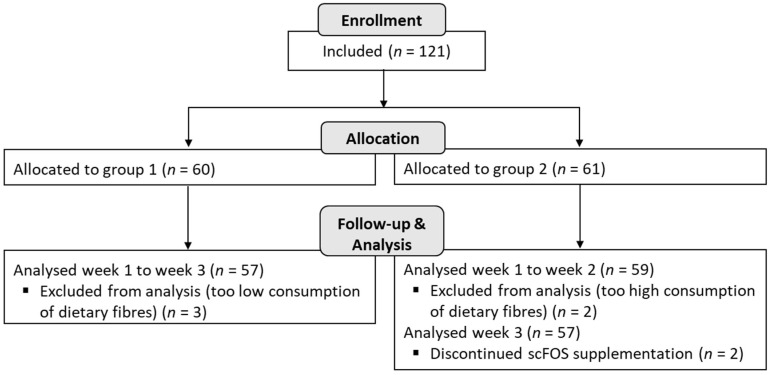
Flow diagram.

A total of 116 subjects (*n* = 57 in group 1 and *n* = 59 in group 2) were analysed in the study, and among them, 114 subjects completed the trial until the end of the 3 weeks (2 subjects did not complete the product consumption during the last week in group 2) (Figure 2).

The subjects were males and females (sex ratio 39.7/60.3), healthy, with a mean age of 36.9 ± 7.9 years (ranging from 18 to 50 years), a mean weight of 74.8 ± 17.9 kg, and a mean body mass index (BMI; weight/(height)^2^) of 25.7 ± 5.3 kg/m^2^. At baseline, all subjects presented a regular stool frequency with a mean value of 8 ± 3.6 stools per week corresponding to 1.2 stools/day (Table 1).

The tolerance of the product was evaluated through the declaration of non-gastrointestinal inconvenience. Four non-gastrointestinal events were reported during the trial. Two adverse events were declared in group 1 at 30 g/d scFOS. They were urinary tract infection and menstrual disorder. Two other events were recorded in group 2 at 40 g/d scFOS, declared as acne and weight loss. These non-gastrointestinal events were considered unrelated to the treatment.

Overall, the mean compliance of the product was higher than 90%, with the lowest compliance for the dose 20 g/d (90.3%) and the highest compliance for the dose of 40 g/d (95.6%). These are very high scores, showing that the scFOS are very well accepted.

**Figure 3:**
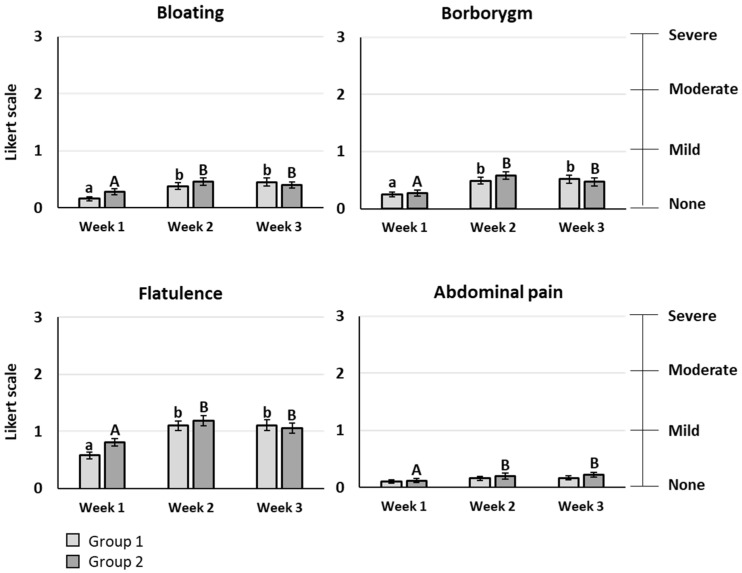
Severity of gastrointestinal symptoms evaluated using Likert scale with four items: bloating, borborygmi, flatulence, and abdominal pain. Values are expressed as means ± SEM. Light grey bars correspond to group 1 and dark grey bars correspond to group 2. Week 1: baseline period without any supplementation; week 2: supplementation with a low dose of scFOS (15 or 20 g/d); week 3: multiplication of scFOS dose by 2 in each group. a, b: *p* < 0.05 in group 1; A, B: *p* < 0.05 in group 2. No significant effect between groups: *p* > 0.05.

The primary outcome of the study was the gastrointestinal comfort evaluated by eight symptoms rated using a Likert scale with 4 items (0: none, 1: mild, 2: moderate, 3: severe symptom). For bloating, borborygmi, flatulence, and abdominal pain, a significant increase of the mean score was observed after scFOS consumption, whatever the dose tested (*p* < 0.05). However, the mean score stayed lower than 1, meaning that the symptom rating remained between “no symptom” and “mild symptom”, except for flatulence, reaching a maximal mean score of 1.19 for 20 g/d (Figure 3). Heartburn, acid reflux, nausea, and vomiting had very low mean scores below 0.1, without any significant difference between the baseline period and the scFOS consumption periods. No significant difference was observed between the different doses of scFOS tested for all gastrointestinal symptoms.

The mean value of stool consistency after scFOS intake was significantly improved compared to the baseline period whatever the scFOS dose tested (*p* < 0.05), and an increase between 20 and 40 g/d was also observed (*p* < 0.05). Nevertheless, the mean scores were between 3 and 5 on the Bristol stool scale during the three weeks of the trial, meaning that no diarrhoea appeared up to 40 g/d scFOS (Figure 3).

**Figure 4:**
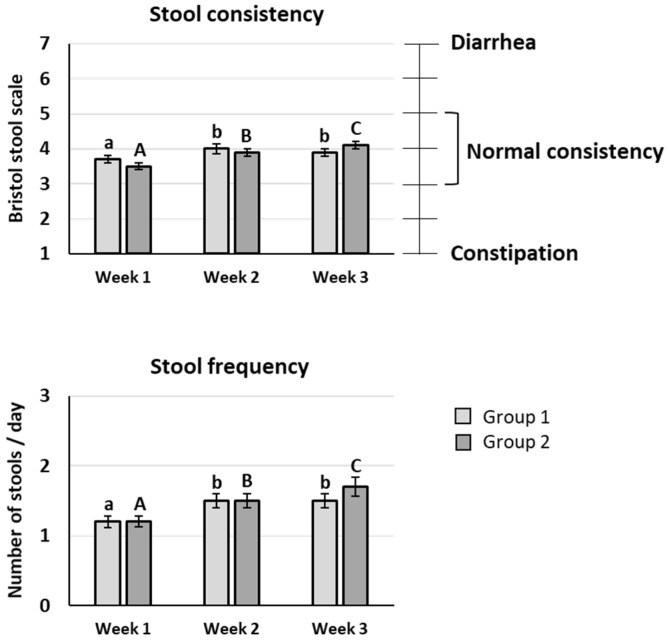
Stool consistency evaluated using Bristol stool scale and stool frequency. Values are expressed as means ± SEM. Light grey bars correspond to group 1 and dark grey bars correspond to group 2. Week 1: baseline period without any supplementation; week 2: supplementation with a low dose of scFOS (15 or 20 g/d); week 3: doubling of scFOS dose in each group. a, b: *p* < 0.05 in group 1; A, B, C: *p* < 0.05 in group 2. No significant effect between groups: *p* > 0.05.

The subjects presented a mean stool frequency comprising between 1 and 2 stools per day, with a significant increase of stool frequency after scFOS intake at 40 g/d vs. 20 g/d (*p* < 0.05) (Figure 4).

At day 14, 78.9% subjects in group 1 and 64.9% in group 2 evaluated their satisfaction linked to gastrointestinal comfort as ‘quite satisfied’ to ‘very satisfied’ on the Likert scale. At day 21 (two-fold increase in scFOS intakes), the degree of satisfaction was similar: 75.5% subjects were quite satisfied to very satisfied with their gastrointestinal comfort in group 1, and 64.9% in group 2 with the highest scFOS dose. Concerning the cofounding factors, no significant change in the PNNS and GPAQ questionnaires were detected. In particular, the fibre consumption remained identical.

## 4. Discussion

The objective of this study was to evaluate the gastrointestinal tolerance of prebiotic fibre scFOS from sugar beet at intakes up to 40 g per day, in a large population of healthy volunteers.

The population of 116 healthy subjects aged from 18 to 50 years included in this study can be considered as representative of a normal European population, with a BMI of 25.7 kg/m^2^ and an average fibre consumption (assessed following the food questionnaire developed by the French public health watch institute (INVS)). The main conclusion of the study is that scFOS are very well tolerated up to 40 g per day. Although a statistically significant difference versus the baseline period was found after scFOS intake, all symptoms were very mild from a clinical perspective, with a maximum mean score of 1.19 obtained for flatulence on a four-item Likert scale. In addition, the mean value of stool consistency stayed normal after scFOS intake, between 3 and 5 on the Bristol stool scale, confirming that no significant clinical change on stool consistency appeared with scFOS consumption up to 40 g/d.

These results corroborate the excellent digestive tolerance found in an older study with few subjects (*n* = 16) whose lifestyle habits and dietary consumption patterns have evolved over the past 25 years. This previous study evaluated the effects of a gradual increase in scFOS intake on the appearance of gastrointestinal symptoms, with the determination of the threshold dose that should not be exceeded [18]. In this study, the first mild symptoms to occur in some subjects were excessive flatulence (>30 g of scFOS), and then borborygmi and bloating (>40 g of scFOS). Abdominal cramps and diarrhoea may only be expected for high dosages of 50 g or more per day of scFOS [18]. Interestingly, the threshold dose, based on the appearance of diarrhoea or a severe gastrointestinal symptom or when the subject did not want to consume more product, was very high and corresponded to 54.3 g after the occasional consumption of scFOS and 58 g after the regular consumption of scFOS [18]. 

In our study, the multiplication of the scFOS dose by two after one week consumption did not induce any increase of gastrointestinal symptoms suggesting a good acceptability and digestive tolerance of high doses following an adaptative period at a lower dose. Stool consistency and stool frequency were slightly increased after the second week of scFOS intake, when the dose was multiplied, but stayed at normal levels. Consequently, we demonstrated that scFOS is well tolerated up to 40 g/d as dietary supplement in subjects already consuming fibres in their usual diet. 

Many factors affect the gastrointestinal acceptability of foods containing prebiotic fibres, including the physico-chemical properties of the fibre, notably the chain length. Several studies have evaluated the digestive tolerance of inulin-type fructans with different DP. A study compared the effects of inulin on the gastrointestinal tolerance of healthy subjects at different doses: 3.75, 8.75, and 16.25 g per day. With the highest dose of 16.25 g of inulin, painful symptoms occurred, such as abdominal cramps, accompanied by bloating, flatulence, and gastrointestinal rumbling [19]. In another study, the gastrointestinal tolerance of inulin (10 g versus 20 g) was assessed in healthy volunteers and showed no significant difference between 10 g of inulin and a placebo, whereas gastrointestinal tolerance evaluation confirmed that 20 g of inulin is significantly less well tolerated than 10 g [20]. In the same way, with 20 g of oligofructose, an increase of flatulence and intestinal bloating was seen in healthy subjects as well as a tendency to higher scores for abdominal pain and cramps, both considered intolerable effects [21]. Bruhwyler et al. (2009) investigated the effects of the length of inulin chains (DP) on digestive tolerance from 5 to 20 g/d. All inulin products tended to increase digestive symptoms whatever the dose, but the change was only significant for inulin with longer chains at 20 g/d, and a significant difference was observed between inulin with shorter chains (DP 2–20) and inulin with longer chains (DP 2–60). This suggests that shorter chain oligosaccharides may be better tolerated than longer ones [22]. Overall, studies on inulin-type fructans consumption described that intakes below 10 g may cause mild gastrointestinal symptoms, intakes between 10 and 15 g of inulin per day may be tolerated by most individuals with only mild effects, and intakes of 15 and 20 g per day may increase the occurrence and severity of the symptoms with sometimes the appearance of intolerable gastrointestinal symptoms [20,22,26,27,28,29,30]. These data on inulin products suggest that the maximum digestive tolerance of inulin and oligofructose from inulin is 20 g/d, thus two times less than the digestive tolerance of prebiotic fibres with shorter chains like scFOS, tested in our study at 40 g/d, as demonstrated now by two specific studies above 20 g/d [18].

These results on the digestive tolerance of scFOS from sugar beet are important information for health care professionals to provide dietary advice to their patients related to their intakes of dietary fibres. They can also be useful for the food industry R&D department to guide them in the formulation of products enriched with dietary fibres, contributing to the increase of fibre intakes in the population. Indeed, according to the European Food Safety Authority (EFSA), based on the available evidence on bowel function, dietary fibre intakes of 25 g/day are considered adequate for normal laxation in adults [5]. According to recent consumption surveys in various member states, fibre intakes are below recommendations, ranging between around 10 and 24 g per day [4]. Improvement efforts on the nutritional profile of food should therefore also focus on increasing fibre content, either through ingredients rich in fibres or through the addition of dietary fibres, while taking into account digestive tolerance data.

## 5. Conclusions

Because of the low fibre intake in the European population, below the recommendations, functional prebiotic fibres such as short-chain fructo-oligosaccharides are being added to foods. Thanks to their very short chains (DP 3–5) and unique composition, scFOS prebiotic fibres (Actilight^®^) are well tolerated up to 40 g/d as a regularly consumed supplement, much better tolerated than other types of inulin-type fructans with longer chains (DP 3–60). Gastrointestinal symptoms were only reported as mild symptoms, and most subjects were satisfied with their intestinal comfort following high doses of scFOS. Digestive tolerance data should be considered when fibres are supplemented in processed foods. Thus, scFOS are appropriate for the formulation of food products to increase dietary fibre intakes.

## Figures and Tables

**Table 1:** Baseline demographic characteristics of the subjects divided in two groups.

Title 1	All Subjects (*N* = 116)	Group 1 (*N* = 57)	Group 2 (*N* = 59)
Age (years)	36.9 ± 7.9	35.8 ± 8.0	37.9 ± 7.8
Sex			
-Male (%)	39.7	40.4	39
-Female (%)	60.3	59.6	61
Weight (kg)	74.8 ± 17.9	75.4 ± 19.4	74.2 ± 16.5
BMI (kg/m^2^)	25.7 ± 5.3	26.1 ± 5.9	25.4 ± 4.6
Stool frequency (nb/day)	1.2 ± 0.5	1.2 ± 0.4	1.2 ± 0.5

Values are expressed as means ± standard deviation or %. BMI: body mass index; Group 1: group supplemented with 15 g/d scFOS during week 2 and 30 g/d scFOS during week 3; Group 2: group supplemented with 20 g/d scFOS during week 2 and 40 g/d scFOS during week 3.

## Data Availability

The data that support the findings of this study are openly available from the corresponding author upon reasonable request.
